# Airway smooth muscle proliferation and survival is not modulated by mast cells

**DOI:** 10.1111/j.1365-2222.2009.03423.x

**Published:** 2010-02

**Authors:** D Kaur, F Hollins, R Saunders, L Woodman, A Sutcliffe, G Cruse, P Bradding, C Brightling

**Affiliations:** Department of Infection, Inflammation and Immunity, Institute for Lung Health, University of LeicesterLeicester, UK

**Keywords:** airway smooth muscle, apoptosis, asthma, mast cells, necrosis, proliferation, survival

## Abstract

**Background:**

Airway smooth muscle (ASM) hyperplasia and mast cell localization within the ASM bundle are important features of asthma. The cause of this increased ASM mass is uncertain and whether it is a consequence of ASM–mast cell interactions is unknown.

**Objective:**

We sought to investigate ASM proliferation and survival in asthma and the effects of co-culture with mast cells.

**Methods:**

Primary ASM cultures were derived from 11 subjects with asthma and 12 non-asthmatic controls. ASM cells were cultured for up to 10 days in the presence or absence of serum either alone or in co-culture with the human mast cell line-1, unstimulated human lung mast cells (HLMC) or IgE/anti-IgE-activated HLMC. Proliferation was assessed by cell counts, CFSE assay and thymidine incorporation. Apoptosis and necrosis were analysed by Annexin V/propidium iodide staining using flow cytometry and by assessment of nuclear morphology using immunofluorescence. Mast cell activation was confirmed by the measurement of histamine release.

**Results:**

Using a number of techniques, we found that ASM proliferation and survival was not significantly different between cells derived from subjects with or without asthma. Co-culture with mast cells did not affect the rate of proliferation or survival of ASM cells.

**Conclusion:**

Our findings do not support a role for increased airway smooth proliferation and survival as the major mechanism driving ASM hyperplasia in asthma.

*Cite this as*: D. Kaur, F. Hollins, R. Saunders, L. Woodman, A. Sutcliffe, G. Cruse, P. Bradding and C. Brightling, *Clinical & Experimental Allergy*, 2010 (40) 279– 288.

## Introduction

Asthma is a chronic inflammatory lung disease that leads to significant morbidity and mortality and its prevalence is increasing [1]. Asthma is characterized by the presence of variable airflow obstruction, airway inflammation, airway hyperresponsiveness (AHR) and airway remodelling [2]. Mast cell microlocalization within the airway smooth muscle (ASM) bundle is a characteristic feature of asthma and is related to the severity of AHR [3–7].

The features of airway remodelling include deposition of collagen in the lamina reticularis beneath the epithelial basement membrane, increased numbers of subepithelial myofibroblasts, and increased glandular and ASM mass [7–9]. The increased ASM mass may be because of a combination of both ASM hyperplasia [8] and hypertrophy, which has been shown to increase with disease severity and is associated with fixed airflow obstruction [8–10]. The cause of ASM hyperplasia in asthma is unknown, but may be a consequence of increased ASM proliferation or survival either because of intrinsic abnormalities in the ASM or because of interactions with the local inflammatory environment. Reports have described increased proliferation in *ex vivo* asthmatic ASM cells [11], which has been attributed to alteration in mitochondrial biogenesis [12]. However, this observation has not been replicated in airway-derived myofibroblasts [13] and many studies have been unsuccessful in demonstrating increased ASM proliferation *in vivo* [9, 10, 13, 14]. Mast cells localized within the ASM bundle have the potential to secrete a plethora of pro-inflammatory mediators, cytokines and proteases through their chronic activation by IgE, allergens and many other diverse non-immunological stimuli [15, 16]. Tryptase, an important mast cell-derived protease, is a potent stimulus for both DNA synthesis and cell proliferation in ASM cells [4], while other studies have shown that chymase another mast cell protease can have a profound effect on ASM cell function and airway remodelling [17]. However, whether whole mast cells modulate ASM proliferation or survival is unclear.

We hypothesized that: (1) there are intrinsic differences between ASM from asthmatics and non-asthmatics with increased proliferation and survival in asthma and (2) these differential responses are modulated by interactions with mast cells. To test our hypothesis, we assessed proliferation, apoptosis and necrosis of ASM cells from subjects with and without asthma, both alone and in co-culture with mast cells.

## Methods

### Subjects

Asthmatic subjects and non-asthmatic controls were recruited from Leicester, UK. Subjects with asthma had a consistent history and objective evidence of asthma, as indicated by one or more of the following: (i) methacholine AHR [PC_20_ forced expiratory volume in 1 s (FEV_1_)<8 mg/mL]; (ii) >15% improvement in FEV_1_ 15 min after administration of 200 μg of inhaled salbutamol or (iii) >20% of maximum within-day amplitude from twice daily peak expiratory flow measurements over 14 days. Severity of asthma was defined by Global initiatives for asthma (GINA) treatment steps I–V [18]. The study was approved by the Leicestershire Research Ethics Committees and all patients gave their written informed consent.

### Airway smooth muscle isolation and culture

Pure ASM bundles in bronchial biopsies were obtained from fibreoptic bronchoscopy (*n*=12, 11 subjects with asthma, one non-asthmatic subject) and additional airways isolated from lung resection (*n*=11). Subjects undergoing lung resection with moderate–severe airflow obstruction were excluded. Only one of the 12 non-asthmatic subjects had lung function impairment (FEV_1_% predicted <80%) and airflow obstruction (FEV_1_/FVC<70%). Clinical characteristics were as shown in Table 1. Primary ASM were cultured in Dulbecco's modified Eagle's medium (DMEM) supplemented with 10% fetal calf serum (FCS), 4 mm l-glutamine, 100 U/mL penicillin, 100 mg/mL streptomycin and 0.25 μg/mL amphotericin (Invitrogen, Paisley, Scotland, UK). ASM characteristics were determined by immunofluorescence, flow cytometry and light microscopy with α-smooth muscle actin–FITC direct conjugate and myosin indirectly conjugated with FITC (Sigma, Poole, UK). ASM purity was 94(1.5)% and cells were used between passage 2 and 5.

**Table 1:** Clinical characteristics of airway smooth muscle donors

	Asthma GINA I=3; II=2; IV=4; V=2	Controls
Number	11	12
Gender, M/F	6/5	6/6
Age (years)	47 (3)	65 (4)
FEV_1_ (L)	2.5 (0.2)	2.1 (0.2)
FEV_1_% predicted	82 (6)	90 (5)
FEV_1_/FVC (%)	72 (2)	77 (2)
Inhaled corticosteroids (*n*)	8	1
Long acting beta-agonist (*n*)	6	0
Oral corticosteroids (*n*)	2	0

Mean (SEM).

GINA, Global initiatives for asthma; FEV_1_, forced expiratory volume in 1 s; FVC, forced vital capacity.

### Mast cells

Human lung mast cells (HLMC) (*n*=17) were obtained from lung resection surgery by positive immunomagnetic selection and cultured as described previously [19]. Final mast cell purity was >99% and viability was >98%. The human mast cell line (HMC-1) was a generous gift from Dr. J. Butterfield (Mayo Clinic, Rochester, MN, USA) and was maintained in Iscove's modified DMEM.

### Non-asthmatic and asthmatic ASM cell assessment

#### Cell counts

ASM cells were seeded at a density of 5 × 10^4^ cells in 60 mm tissue culture dishes (1.8 × 10^3^cells/cm^2^); serum deprived for 24 h in serum-free ITS media (Sigma), and then exposed to 10% FCS over 10 days before harvesting with trypsin and counting using trypan blue.

#### Carboxyfluorescein succinimidyl ester quantification

ASM cell proliferation was also assessed using the CFSE Proliferation Kit (Invitrogen). ASM cells were seeded into 60 mm tissue culture dishes and serum deprived for 24 h as per cell counts above. Cells were then labelled with 2.5 μm carboxyfluorescein succinimidyl ester (CFSE) for 15 min in phosphate-buffered saline (PBS), before incubation in 10% FCS media for a further 30 min to allow cleavage of the acetate groups to yield highly fluorescentCFSE. They were then incubated in 10% FCS at 37 °C over 10 days. CFSE fluorescence was analysed using single colour flow cytometry (BD FACSCanto; BD Pharmingen, Oxford, UK).

#### Thymidine assay

ASM cells were seeded in quadruplicate in 96-well flat-bottomed plates at a density of 1 × 10^3^cells/well in 10% FCS media over 48 h. ASM cells were then serum deprived in serum-free media for 24 h and pulsed with 1 μ*C*_i_^3^H-thymidine (Amersham Pharmacia Biotech Ltd., Buckinghamshire, UK) in 10% FCS media for 24 h. Cells were washed with PBS and then precipitated with TCA for 1 h on ice and fixed with ethanol overnight. Cells were then lysed with 200 μL SDS and added to 10 mL of scintillant. The solution was mixed and counts per minute (CPM) were assessed on the TRI-CARB® scintillation analyser (model 1500, Packard, Pangbourne, Berks, UK).

#### Cell metabolic activity assay

ASM cells were seeded in triplicate in 96-well flat bottomed plates at 1 × 10^3^ cells/well in 10% FCS media over 48 h at 37 °C and then serum deprived for 24 h. The CellTiter 96 Aqueous one solution with the tetrazolium compound MTS [3-(4,5-dimethylthiazol-2-yl)-5-(3-carboxymethoxyphenyl)-2-(4-sulfophenyl)-2H tetrazolium] solution (Promega, Southampton, UK) was then added to each well and incubated at 37 °C for 3.5 h. MTS is chemically reduced by the cells to formazan, the absorbance of which at 490 nm gives a measure of the dehydrogenase enzyme activity found in metabolically active cells.

### ASM/mast cell co-culture

As above ASM cells were seeded at a density of 5 × 10^4^ cells then serum deprived for 24 h. ITS media was then removed and replaced with either fresh ITS or 10% FCS media ± HMC-1 cells, goat polyclonal anti-human IgE (1/500) (Sigma), HLMC alone or HLMC that had been sensitized with 2.4 μg/mL human myeloma IgE (Calbiochem-Novabiochem, Nottingham, UK) and then activated with goat polyclonal anti-human IgE (1/500) in co-culture. The ASM cells alone and with mast cells were cultured over 10 days without refeeding. The mast cells were added at a 1 : 4 (mast cell : ASM) ratio to reflect our *in vivo* observations. After 10 days, the supernatant was collected and the cell pellet was lysed in sterile de-ionized water. ASM cell monoculture control cell counts were established in parallel with mast cell co-cultures using the Kimura staining, which readily differentiates red metachromatic mast cells from unlabelled ASM cells.

#### Quantification of histamine release by mast cells

Histamine release by mast cells in co-culture was measured by sensitive radioenzymatic assay based on the conversion of histamine to methylhistamine in the presence of the enzyme histamine-*N*-methyltransferase as described previously [17]. Control HLMC were sensitized with IgE (2.4μg/mL) for 1 h, washed and then activated with 1/500 anti-IgE for 1 h.

#### Quantification of ASM death by flow cytometry

ASM cultured alone was harvested using Accutase and the percentage of apoptotic or necrotic cells was assessed by suspending the ASM cells in 1 × Annexin binding buffer containing FITC-conjugated Annexin V [(1 μL/200 μL) binding buffer]±propidium iodide (PI) (0.5 μg/mL; BD Pharmingen), before analysis on the BD FACSCanto flow cytometer. Three colour flow cytometry was conducted with ASM co-cultured with HMC-1 or HLMC. The co-cultured cells were stained with mAb antibody against CD117 (clone:YB5.B8) (BD Pharmingen) and followed by allphophycocyanincon (APC) secondary antibody to identify the HLMC. This allowed HLMC to be gated out of the ASM cell analysis and then stained as above with Annexin and PI. Appropriate isotype controls were used.

#### Morphological detection of ASM apoptosis

Nuclear morphology of ASM was assessed by 4′,6-diamidino-2-phenylindole (DAPI) staining. For these experiments, ASM were seeded at a density of 3 × 10^3^ cells/well into eight-well chamber slides and incubated at 37 °C for 48 h. The media was then replaced with ITS media and incubated at 37 °C over 3 days. After 3 days of ASM cell serum deprivation, ITS media was removed and replaced with fresh ITS media±HMC-1 cells or HLMC at 1 : 4 ratio. The ASM cells were co-cultured with the mast cells over 10 days. ASM cells co-cultured with HLMC were stained with directly conjugated mAb against RPE-CD117 and indirectly labelled with secondary NorthenLights RPE secondary antibody (R&D Systems, Abingdon, UK). The cells were then counterstained with DAPI and mounted with photo bleach retardant mounting medium. CD117 was used to identify the HLMC in the presence of ASM cells. ASM cells cultured alone were stained with DAPI alone and mounted. For each ASM mono- or co-culture, six random high-powered fields (hpf) were examined for morphologic features of apoptosis such as nuclear condensation and fragmentation.

## Statistical analysis

Statistical analysis was performed using GraphPad Prism 4 (GraphPad, San Diego, CA, USA). Data are presented as mean±SEM and log normally distributed data was presented as geometric mean±log SEM or ±95% confidence interval. Comparisons across groups were performed by anova and Tukey's *post hoc* test, and between groups by paired and unpaired *t*-tests as appropriate. Differences were considered significant when *P*<0.05.

## Results

### Non-asthmatic and asthmatic ASM cell proliferation and metabolic activity

**Fig. 1:**
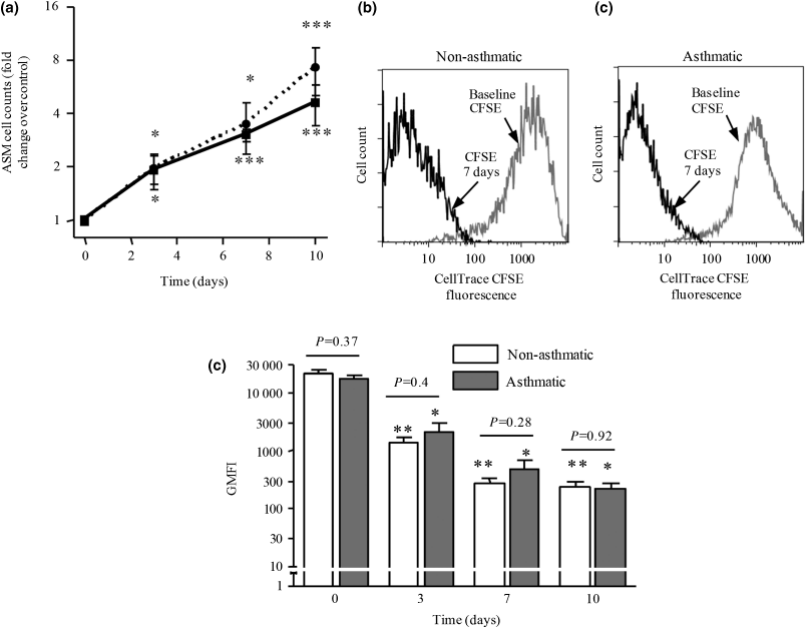
(a) Number of non-asthmatic (black line, *n*=7) and asthmatic (dashed line, *n*=7) airway smooth muscle (ASM) cells in response to exposure to 10% fetal calf serum (FCS) media over 10 days (data presented as mean fold change compared with control ± SEM). Flow cytometric histogram illustrating carboxyfluorescein succinimidyl ester (CFSE) fluorescence in (b) non-asthmatic and (c) asthmatic ASM cells in response to exposure to 10% FCS media over 7 days. (d) Non-asthmatic (*n*=4) and asthmatic (*n*=4) ASM cell proliferation was observed over 10 days. Data are presented as geometric mean±logSEM. Comparisons were made to CFSE staining at day 0 for non-asthmatic or asthmatic ASM cells. ^*^P<0.05, ^**^P<0.01, ^***^P<0.001 for time-points compared with baseline and the *P*-values for comparisons between asthma and controls are as shown.

After 3, 7 and 10 days in culture, there was a significant difference in ASM cell number compared with baseline counts (day 0) in both non-asthmatic [day 10 mean fold difference (95% CI); 4 (2.43–6.52); *P*=0.0005; *n*=7] and asthmatic ASM cells [day 10 mean fold difference (95% CI); 5.86 (3.16–10.84); *P*=0.0004; *n*=7; Fig. 1a]. However, there was no significant difference in the rate of ASM proliferation between non-asthmatic and asthmatic ASM cells (Fig. 1a). There was no relationship between ASM proliferation and asthma severity (data not shown).

Example flow cytometry histograms of CFSE staining of asthmatic and non-asthmatic cells exposed to 10% FCS are shown (Figs 1b and c). The geometric mean fluorescence intensity (GMFI) markedly decreased from baseline (day 0) after 3, 7 and 10 days confirming that the ASM cells were actively proliferating. However, we did not observe differences between asthmatic (*n*=4) and non-asthmatic ASM cells (*n*=4; Figs 1b–d). Similarly we were unable to detect a difference in the tritiated thymidine incorporation over 24 h between ASM from asthmatics (*n*=4) and controls [*n*=4; mean difference (95% CI); 78 (−147 to 303) cpm; *P*=0.43).

The absorbance by formazan seen at 490 nm in the MTS assay was increased in ASM cells (*n*=8) in media supplemented with 10% FCS compared with ASM cells in serum-free media over 24 h (data not shown). However, there was no difference between non-asthmatic (*n*=4) and asthmatic (*n*=4) ASM cells over 24 h [OD mean difference (95% CI); −0.03 (−0.49–0.44)%; *P*=0.9] and 72 h [OD mean difference (95% CI); 0.14 (−0.21–0.48)%; *P*=0.37].

### Non-asthmatic and asthmatic ASM cell proliferation in co-culture

**Fig. 2:**
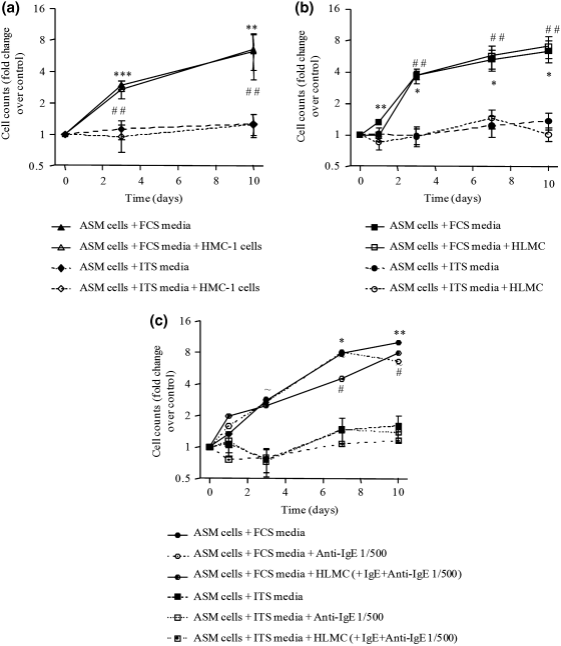
(a) Number of airway smooth muscle (ASM) cells in response to exposure to 10% fetal calf serum (FCS) media ± human mast cell line (HMC-1) cells (*n*=6) or ITS media ± HMC-1 cells over 10 days (*n*=7). (b) ASM cell counts in response to exposure of 10% FCS media ± HLMC (*n*=3) or ITS media ± HLMC (*n*=7) over 10 days. (c) Number of ASM cells in response to either 10% FCS or ITS media alone ±1 : 500 anti-IgE or co-cultured with activated HLMC (IgE/anti-IgE activation, *n*=4 ASM donors and four HLMC donors). Comparisons were made to control (day 0) within ASM status group using the one sample *t*-test. Data presented as fold change geometric mean±logSEM. (^*^), ASM alone with 10% FCS; (^#^), ASM ± HMC-1 cells or HLMC in 10% FCS; (∼), ASM ± HLMC (IgE-Anti-IgE 1/500 activated). ^*^P<0.05, ^**^P<0.01, ^##^*P*<0.01, ^***^P<0.001, ∼*P*<0.05 for time-points compared with baseline.

ASM cells proliferated in the presence of 10% FCS, but not ITS (Figs 2a–c). HMC-1 cells did not affect the ASM cell count in 10% FCS (*n*=6) or ITS media (*n*=7) over 3 or 10 days (Fig. 2a). There were no differences between asthmatic or non-asthmatic cells (data not shown). Similarly, co-culture of HLMC with ASM in 10% FCS (*n*=3) or ITS media (*n*=3) did not affect the cell number over 10 days (Fig. 2b).

Co-culture of ASM with IgE/anti-IgE activated HLMC did not affect ASM proliferation over 10 days in the presence of ITS media or media supplemented with 10% FCS (*n*=4; Fig. 2c).

### Histamine release in HLMC and ASM co-culture

**Fig. 3:**
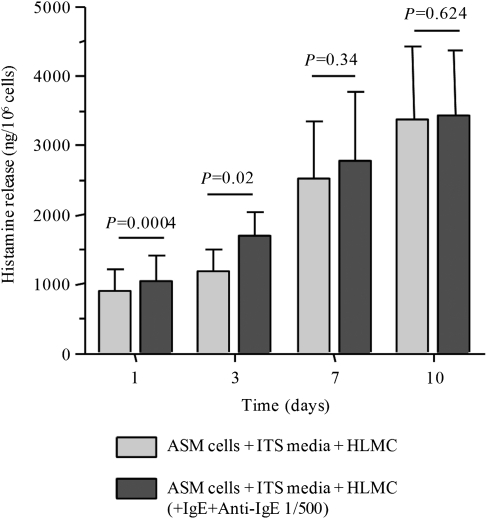
Airway smooth muscle (ASM) cells (*n*=4) were co-cultured with un-activated human lung mast cells (HLMC, *n*=4) alone and activated HLMC (*n*=4, IgE/Anti-IgE) over 10 days in ITS media. Histamine concentrations were measured in culture supernatants and corrected for mast cell number. Constitutive histamine release was demonstrated with un-activated HLMC co-culture, which was augmented in IgE/anti-IgE activated HLMC over 1 and 3 days. Data presented as mean±SEM; *P*-values are as shown.

HLMC alone (*n*=4) released significantly more histamine with IgE/anti-IgE activation at day 0 (918±101 ng/10^6^ cells) when compared with un-activated HLMC (495±85 ng/10^6^ cells; *P*=0.001). In co-culture, histamine was readily measured at all time-points and increased over time with concentrations at day 7 (*P*=0.03) and 10 (*P*=0.02) significantly higher than day 1 (Fig. 3). Histamine release by HLMC co-cultured with ASM cells and IgE/anti-IgE activated was increased compared with unstimulated co-cultured HLMC after 1 and 3 days (Fig. 3).

### ASM apoptosis and necrosis in co-culture

**Fig. 4:**
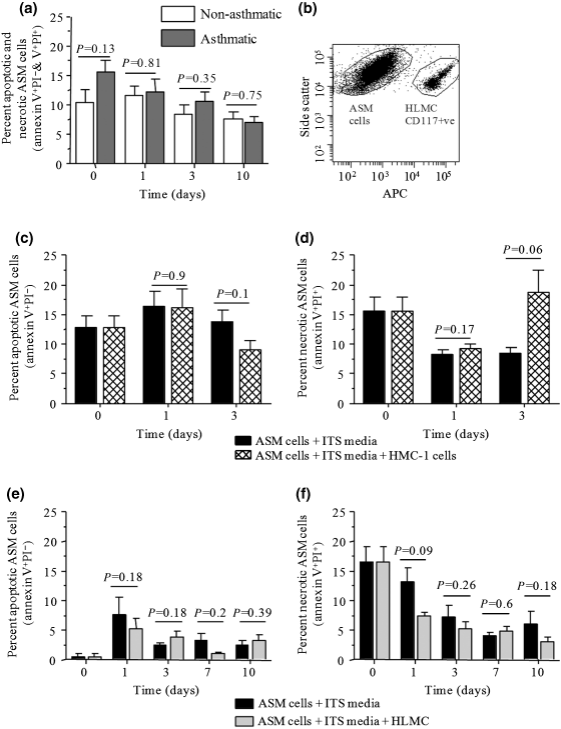
Apoptosis and necrosis was assessed by flow cytometry with FITC-Annexin V/PI binding in airway smooth muscle (ASM) cells alone or co-cultured with human mast cell line (HMC-1) or human lung mast cells (HLMC) labelled with CD117-APC. (a) Percentage apoptotic ASM cells revealed by Annexin V^+^/PI^−^ and necrotic cells revealed by Annexin V^+^/PI^+^ cells of non-asthmatic (*n*=6) and asthmatic (*n*=4) ASM cells. (b) Representative flow cytometry dot plots illustrating day 7 co-culture of ASM cells and HLMC labelled with CD117-APC. (c) Percentage of apoptotic ASM cells ± HMC-1 cells and (d) percentage of necrotic ASM cells ± HMC-1 cells (*n*=7 paired *t*-test), (e) Percentage of apoptotic ASM cells ± HLMC cells and (f) percentage of necrotic ASM cells (*n*=3). Data presented as mean±SEM; *P*-values are as shown.

A combination of Annexin V staining and PI staining was used to identify ASM cell apoptosis and necrosis in the various culture conditions. Annexin V^+^/PI^−^ cells were classed as early apoptotic and Annexin V^+^/PI^+^ cells as late apoptotic/necrotic [20]. There was no difference in ASM cell percentage apoptosis and necrosis between ASM derived from subjects with (*n*=4) or without asthma (*n*=6; Fig. 4a). In co-culture CD117-APC labelled mast cells were distinguished from ASM by flow cytometry as shown (Fig. 4b). The proportion of apoptotic and necrotic cells was not significantly affected by the presence of HMC-1 cells over 3 days (*n*=7) or by HLMC over 10 days (*n*=3; Figs 4c–f).

**Fig. 5:**
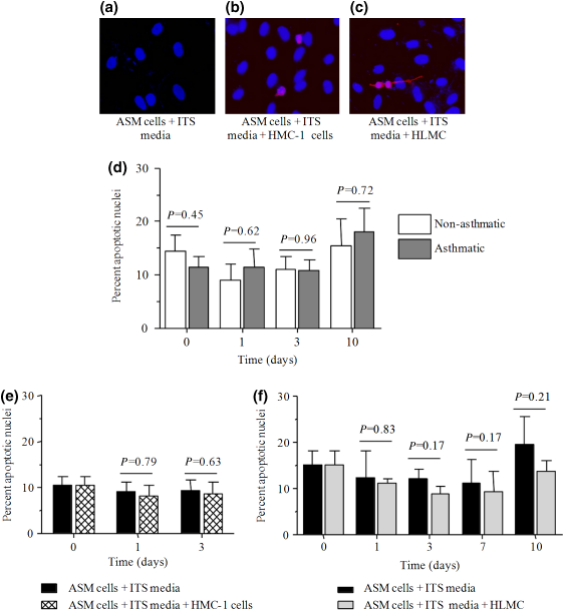
(a) Representative micrographs at day 3 showing 4′,6-diamidino-2-phenylindole (DAPI) staining of airway smooth muscle (ASM) cells in the presence of ITS media alone, (b) in co-culture with human mast cell line (HMC-1) cells [stained with DAPI (blue) and overlaid with anti-CD117 (red)]. (c) ASM cells in co-culture with human lung mast cells (HLMC) cells (stained with DAPI and overlaid with anti-CD117). (d) Percentage of apoptotic nuclei of non-asthmatic (*n*=9) and asthmatic ASM cells (*n*=7) identified by nuclear morphology over 10 days. (e) Percentage of apoptotic nuclei for ASM cells in ITS media ± HMC-1 cells over 3 days (*n*=7) and (f) ASM cells in ITS media ± HLMC over 10 days (*n*=9). Data presented as mean±SEM; *P*-values are as shown.

Example immunofluorescent photomicrographs of mast cells in co-culture with ASM are as shown (Figs 5a–e). Mast cells were identified as CD117+ and excluded from examination of nuclear morphology. ASM nuclear DAPI staining supported the Annexin V data as there was no difference between the percentage apoptotic cells from subjects with (*n*=7) and without asthma (*n*=9; Fig. 5d). ASM cells were unaffected by the presence of HMC-1 cells over 3 days (*n*=7; Fig. 5e) and 10 days for the HLMC (*n*=9; Fig. 5f).

Using both DAPI and Annexin V/PI staining, no difference in the effect of HMC-1 cells was observed between cells derived from asthmatic subjects compared with non-asthmatic controls (data not shown).

## Discussion

We found no differences in proliferation or survival of ASM from subjects with and without asthma. Co-culture of ASM with unstimulated or stimulated mast cells did not modulate ASM proliferation or survival. We therefore reject our hypothesis that there are intrinsic differences in proliferation and survival of asthmatic ASM augmented by the co-localization of mast cells. Our findings do not support a role for increased ASM proliferation and survival as the major mechanism driving ASM hyperplasia in asthma.

The view that there is increased proliferation of airway mesenchymal cells in asthma is contentious. Some reports supported this view [11, 12], but more recent work has been unable to replicate these findings [13]. Here we did not identify a difference in proliferation between cells from subjects with and without asthma. The strength of our assertion comes from the consistency in our findings using several techniques and although our control subjects were not all healthy controls, only one out of 12 had spirometric evidence of chronic obstructive pulmonary disease. An alternative explanation for the presence of ASM hyperplasia in asthma is that there is differential survival of ASM cells. We report for the first time the proportion of apoptotic or necrotic ASM cells from subjects with and without asthma. No differences were observed between disease and controls using complementary techniques of Annexin V/PI staining and nuclear morphology. Therefore, in contrast to the consistent observation of intrinsic differences between the synthetic function of ASM from asthmatics compared with non-asthmatics [5, 21–23], we were unable to demonstrate differences in ASM in terms of proliferation or survival.

Importantly, in asthma mast cells are localized to the ASM bundle, which is a consistent feature of this disease [3–7]. Mast cell products such as β-tryptase [4, 24], histamine [25] and LTD_4_ [26], have been implicated in modulating ASM proliferation. We therefore predicted that co-culture of mast cells and ASM would augment proliferation and reduce apoptosis. However, we were unable to demonstrate an effect on ASM proliferation or survival by mast cells even after IgE/anti-IgE stimulation. This illustrates how effects of individual mediators may not reflect whole cell co-culture and underpins the importance of co-culture studies. Interestingly, a recent paper by Ceresa et al. [27] found that ASM proliferation was decreased in 3-D collagen gel vs. 2-D culture. This was associated with de-differentiation of the ASM cells. Co-culture with HMC-1 cells promoted a more ASM phenotype with increased α-smooth muscle actin expression and the re-establishment of the rate of proliferation. This is in keeping with our work that demonstrated mast cells drive ASM differentiation to a more contractile phenotype via autocrine up-regulation of TGF-β1 [28]. Therefore, although we were unable to show that mast cells affect ASM proliferation or survival it remains a possibility that *in vivo* these interactions may have important functional effects which need to be further defined.

Our findings have implications for our understanding of the cause of ASM hyperplasia in asthma. Current dogma suggests that ASM hyperplasia is a consequence of increased ASM proliferation [11]. Our findings do not support a role for proliferation or survival, which suggests that an alternative mechanism may be important in driving ASM hyperplasia such as increased recruitment of ASM or its progenitors [29, 30] to the ASM bundle. This view finds support with increased ASM progenitors in the peripheral blood, lamina propria and most importantly within the ASM bundle of asthmatics [30–32]. We have proposed that the CCL19/CCR7 axis and platelet-derived growth factor may be important in the recruitment of myofibroblasts and fibrocytes, respectively, towards the ASM bundle [29, 30].

One potential criticism of our findings is that we have restricted our study to *ex vivo* primary cultures and have not studied proliferation *in vivo*, and therefore our models may have not reflected the complex interactions with the microenvironment in tissue. However, our confidence in our data is supported by the consistent lack of evidence in favour of increased ASM proliferation *in vivo* [9, 10, 13, 14]. Our view is also strengthened by the lack of an effect on ASM proliferation or survival even following mast cell activation. Interestingly, in co-culture with ASM, mast cells are in an ‘activated state’ evidenced by increased degranulation and constitutive release of histamine [33]. This effect is IgE-independent and suggests that in asthma, ASM will be exposed to high local concentrations of mast cell mediators. Importantly, we have observed other profound effects in whole co-culture without mast cell stimulation [28] and therefore feel confident that we have not overlooked important mast cell mediated effects on ASM survival or proliferation. An alternative explanation for our lack of difference in ASM proliferation between asthma vs. non-asthma maybe related to seeding density. However, ASM and myofibroblast proliferation has previously been reported to be increased in models using both higher [34] and lower seeding densities [35], but also to be unaffected in a study using higher density [36]. Taken together these data suggest that differences in seeding density are unlikely to provide an explanation for the inconsistency between reports. In spite of our inability to identify changes in proliferation and survival of ASM asthmatic, it is important to note that asthma is a heterogeneous condition [37] and therefore, we are unable to exclude the possibility that increased proliferation and survival of ASM may be a feature of some asthmatics.

In conclusion, proliferation and survival of ASM from asthmatics and non-asthmatics is similar. Although mast cell–ASM interactions may play a key role in some important aspects of asthma, mast cell–ASM co-culture does not affect ASM proliferation or survival. ASM proliferation and survival is therefore unlikely to be a key determinant in the development of ASM hyperplasia in asthma.
